# Determination of Mebudipine in Human Plasma by Liquid Chromatography–tandem Mass Spectrometry

**Published:** 2015

**Authors:** Arezoo Asgari, Farzad Kobarfard, Fariborz Keyhanfar, Shohreh Mohebbi, Maryam Noubarani

**Affiliations:** a *Department of Pharmacology and Toxicology, School of Pharmacy, Zanjan University of Medical Sciences, Zanjan, Iran.*; b *Department of Medicinal Chemistry, School of Pharmacy, Shahid Beheshti University of Medical Sciences, Tehran, Iran.*; c *Department of Pharmacology, Faculty of Medicine, Iran University of Medical Sciences, Tehran, Iran.*; d *Department of Medicinal Chemistry, School of Pharmacy, Zanjan University of Medical Sciences, Zanjan, Iran.*

**Keywords:** Mebudipine, Liquid chromatography-mass spectrometry, Human plasma

## Abstract

In previous studies, mebudipine, a dihydropyridine calcium channel blocker, showed a considerable potential to be used in cardiovascular diseases. The aim of the current study was to develop a valid method using reversed-phase high performance liquid chromatography coupled with electrospray ionization mass spectrometry to assay mebudipine in the human plasma. Separation was achieved on a Zorbax Eclipse^®^ C18 analytical column using a mobile phase consisted of methanol/water (90:10, v/v). The flow rate was 0.6 mL/min and carbamazepine was used as an internal standard (IS). This method involved the use of [*M *+Na]^+^ ions of mebudipine and IS at *m*/*z *411 and 259, respectively with the selected ion monitoring (SIM) mode. There were no interfering peaks from endogenous components in blank plasma chromatograms. Standard curves were linear (r^2^>0.99) between 5 to 100 ng/mL. The mean extraction efficiency was about 84% and the limit of quantification for mebudipine was 5 ng/mL in plasma. The coefficient of variation and error at all of the intra-day and inter-day assessments were less than 11%**. **The results indicated that this method is a fast, accurate, sensitive, selective and reliable method for the determination of mebudipine in the human plasma. The assay method has been successfully used to estimate plasma concentration of mebudipine after the oral administration of 2.5 mg tablet in healthy adults.

## Introduction

**Figure 1:**
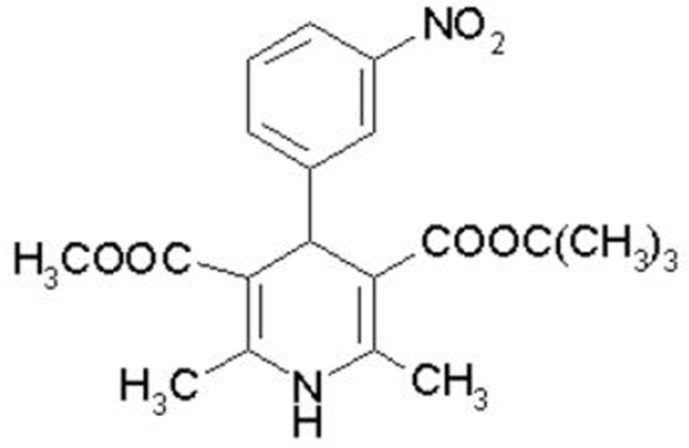
Chemical structure of mebudipine.

Mebudipine [(±)-t-butyl, methyl-1, 4-dihydro-2, 6-dimethyl-4 -(3-nitrophenyl)-3,5-pyridine dicarboxylate] is a dihydropyridine derivative with a calcium channel blocking property (Figure 1) that was first synthesized by Mahmoudian* et al. *in 1997 (1).

Calcium channel blocking property of mebudipine was confirmed in early studies (1, 2). Also mebudipine antagonizes the contractile response of K^+^-depolarized guinea-pig common bile duct to calcium (3). Mebudipine is as potent as amlodipine in inhibition of peak Ca^2+^ currents in differentiated PC12 cells (4).

Mebudipine is a vasoselective and potent blood pressure lowering compound (5). Due to selective and protective effect in ischemic regions (ischemia-selectivity), antioxidant activity against myocardial ischemia-reperfusion injury (6) reduction in the intensity of myocardial ischaemia–reperfusion injury (7) and lack of harmful effects such as reflex tachycardia and heart failure which have sometimes been seen with the older agents (8), mebudipine is candidated to be used in cardiovascular diseases. Previous studies have demonstrated that mebudipine exhibited other effects such as inhibition of phenylephrine-elicited perfusion pressure in isolated kidney from diabetic rats (9,10) and protective effects against glutamate and oxygen–glucose deprivation-induced neurotoxicity (11,12)**.** Also it was reported that mebudipine and its metabolites are not mutagenic on *Salmonella *TA102 (13).

Mebudipine has potential for further development in view of its comparable potency to nifedipine but animal models have shown that mebudipine's oral bioavailability is very low, which is similar to other dihydropyridins (14-17). Slow rates of dissolution and extensive first-pass effects cause the low oral bioavailability of these dihydropyridines. In recent study low oral bioavailability of mebudipine was improved by using phytosolve and phosal-bsed formulation (18). A sensitive and specific analytical method is needed for determination of mebudipine in human after oral administration. Although some Gas Chromatogrphy (GC) methods could provide high sensitivity for the pharmacokinetic study, thermal decomposition of mebudipine under GC condition has been the major problem. High performance liquid chromatography (HPLC) coupled with ultra violet detection offers another possibility for the determination of mebudipine in biological samples, but these methods were limited by low sensitivity, long analysis time or large volume of plasma samples required (19).

Liquid chromatography-mass spectrometry (LC/MS) is now a routine technique that can be applied to a wide range of biological molecules and play an important role in several areas of clinics (20,21) thus the high sensitivity and fast analysis of Liquid Chromatography–tandem Mass Spectrometry (LC/MS-MS) may also benefit in the pharmacokinetic and clinical studies of mebudipine in human. The aim of the present study was to develop a sensitive, simple, fast and reliable LC/ESI-MS method for the determination of mebudipine in human plasma.

## Experimental

*Chemicals*

HPLC grade acetonitrile and methanol (Darmstadt,Germany) were used for the LC/MS analysis. Deionized water was used from a Milli-Q system (Millipore, Bedford, MA, USA). Sodium chloride and acid formic were purchased from Merck (Darmstadt, Germany). Mebudipine was obtained from the laboratory in Iran University of Medical Sciences (Tehran). All other chemicals were of analytical grade.

*Apparatus and chromatographic conditions*

*Liquid chromatography*

Separations were performed on a 150 mm × 4.6 mm OD, 5 μm particle, Agilent Zorbax Eclipse^®^ C18 analytical column. The mobile phase was a mixture of methanol/water (90:10, v/v) and the pH of the mixture was adjusted at 2.6 with formic acid and delivered at a flow rate of 0.60 mL/min. Total run time was 7 min. The wavelength of UV detection was set at 238 and 284 for mebudipine and carbamazepine as internal standard (IS) assay, respectively.

*Mass spectrometry*

**Figure 2:**
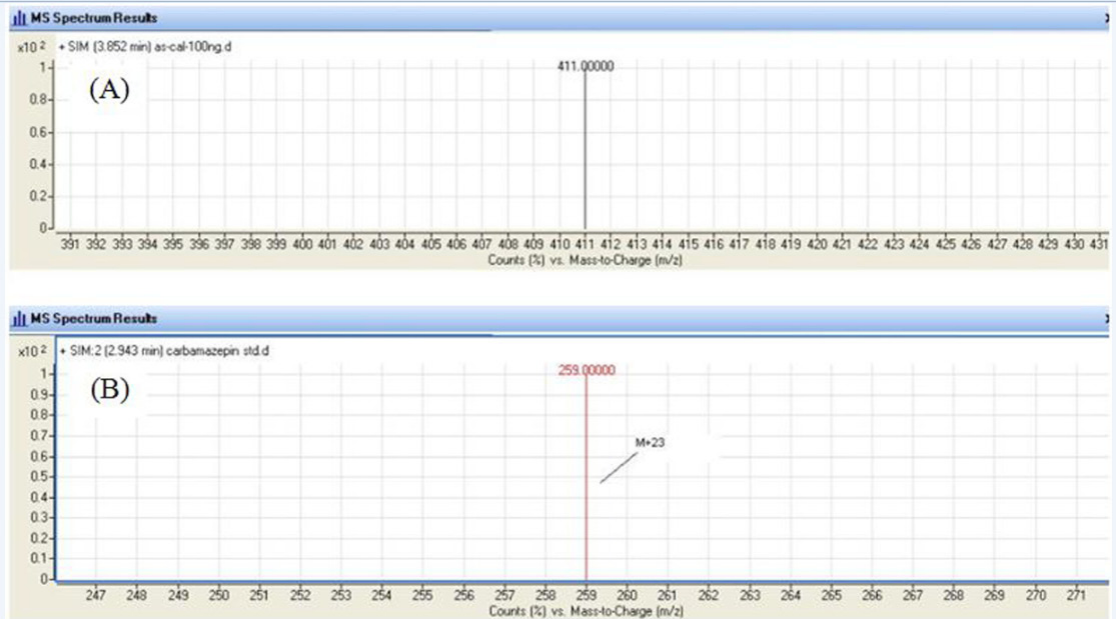
Single Ion Monitoring (SIM) mass spectrum for mebudipine (A) and carbamazepine (B).

An agilent LC/MS-6410 Triple Quadruple mass spectrometer interfaced with electrospray ionization (ESI) ion source was used. Positive selected ion monitoring (SIM) mode and [M +Na]^+^ for both mebudipine (m/z 388→411) and carbamazepine (m/z 236 → 259) were chosen for determination of mebudipine (Figure 2). Optimal MS parameters were as follows: Drying gas (nitrogen) flow rate: 6 L/min, Nebulizing gas adjusted at 15 psi, capillary voltage of 4.0 kV, and desolvation temperature of 300 ºC. Nitrogen was used as the desolvation gas. The data were processed using MassHunter software.

*Plasma sample preparation*

To a 450 µL aliquot of plasma sample in a 2 mL clean tube, 50 µL of IS solution and 500 µL acetonitrile and NaCl were added. The mixture was vortexed for 1 min. After centrifugation at 8000 rpm for 5 min, the upper layer was transferred into an autosampler vial. An aliquot of 20 µL was injected into the LC/MS-MS system for analysis.

*Preparation of standard solutions*

The stock solutions of mebudipine (1 mg/mL) and IS (carbamazepine 500 ng/mL) were prepared in methanol. Working solutions were prepared from the stock solution by sequential dilution with methanol just before use and were protected from light by covering them with aluminum foil.

*Method validation*

The method was validated for selectivity, linearity, precision, accuracy, extraction recovery, matrix effect and stability according to FDA guidance for validation of bioanalytical methods (22).

*Selectivity*

The selectivity was evaluated by comparing chromatograms of different batches of blank plasma obtained from six subjects with those of corresponding standard plasma samples spiked with mebudipine and IS, and those of plasma samples obtained after oral dose of 2.5 mg mebudipine tablet.

*Linearity and LLOQ*

To 400 μL of blank human plasma, 50 μL of working standard of mebudipine was added, yielding final concentrations of 5-100 ng/mL of mebudipine. To this mixture, 50 μL of IS working solution was added to yield IS concentration of 50 ng/mL. Calibration samples were prepared for analysis as described above. Calibration curve was analyzed three times with at least six different concentrations using the same LC conditions as described above. The peak area ratios of drugs to the IS for each of the standard solutions were calculated and plotted as a function of drug concentrations in human plasma. The calibration curves were acceptable only if they had correlation coefficients (r^2^) of 0.99 or greater. The acceptance criterion for each back-calculated standard concentration was 15% deviation from the nominal value except for the lower limit of quantification (LLOQ), which was set at 20% (22). The LLOQ is defined as the lowest concentration on the calibration curve, at which an acceptable accuracy (error) within ±20% and a precision (coefficient of variation, CV) below 20% can be obtained.

*Precision and accuracy*

To examine the accuracy and precision of our method, plasma spiked with three concentrations consisting of LLOQ, middle and high concentrations of the analyte were prepared. Intra-day precision and accuracy were evaluated by analyzing the spiked controls three times a day. This was repeated on three separate days to permit an assessment of inter-day accuracy and precision. Precision was evaluated at each concentration by comparing the values for the coefficient of variation: CV % = (SD/ mean measured concentration) × 100 and accuracy of the assay was determined in terms of % error by dividing the measured concentration minus the expected concentration to the expected concentration × 100.

*Extraction recovery*

The extraction efficiency of mebudipine was determined by analyzing six replicates of plasma samples at three concentration levels of 10, 50 and 100 ng/mL. The recovery was calculated by comparing the peak areas obtained from extracted spiked samples with unextracted equal amount of standards**.**

*Application to human study*

In order to test for utility of the described method for determination of mebudipine concentration after oral administration, mebudipine was administered as a single oral dose in male subjects. Two subjects aged 27-50 years, weighted 54-83 Kg enrolled in the study. All the subjects were healthy and none were taking any medication. The plasma samples were collected up to 6 h after a single oral dose of 2.5 mg mebudipine. Blood samples were collected in heparinized tubes and centrifuged for 15 min at 3000 rpm and the plasma was separated and stored at -80 ºC until analysis. 450 µL plasma sample was spiked with 50 μL IS and processed as mentioned in the extraction procedure section.

## Results and Discussion

*Optimization of* *chromatographic and* *mass spectrometric condition*

Chromatographic conditions were optimized to obtain high sensitivity, good peak shape and short retention time. Composition of the mobile phase was found to be the critical factor for achieving good chromatographic peak shape and resolution. In the present study, a mixture of methanol/water (90:10 v/v, pH adjusted at 2.6) was used as a mobile phase. The selection of carbamazepine as the IS was based on that several substances, such as nifedipine, carbamazepine, phenytoin and amlodipine, were tested as IS. Among these, carbamazepine has been chosen because of its stability and the appropriate elution time. Several extraction solvents including diethyl ether, perchloric acid, zinc sulphate, tert-butyl methyl ether and acetonitril were investigated for the liquid–liquid extraction resulting in low recovery or multiple step in extraction. Acetonitrile was found to be more efficient with extraction recovery of mebudipine about 84%, while the recoveries of other solvents were below 45%. Furthermore, the sample preparation procedure which consists of one-step protein precipitation and direct injection is much simpler than those of other methods. Quality control samples were stable for 1 months if stored frozen at below −80 ºC. It was found that mebudipine was stable at room temperature for 12 h. The standard solutions of mebudipine in mobile phase allowed standing at room temperature 12 h and prepared freshly every day.

In order to select an appropriate ionization mode in LC/MS analysis, the mass spectra were acquired in ESI mode by scanning from 50 to 850 amu. The base peak intensities obtained in positive mode were higher than those obtained in negative mode. The positive ion mass spectra of mebudipine and IS in scan mode were characterized by a Na adduct molecular ion [*M *+Na]^+^ at *m*/*z *411 and 259, respectively as base peaks. Therefore, SIM mode involved selective monitoring of *m*/*z *411 and 259 in the vicinity of retention times for mebudipine and carbamazepine, respectively. The optimized ESI-MS conditions are described in Mass spectrometry section.

**Figure 3:**
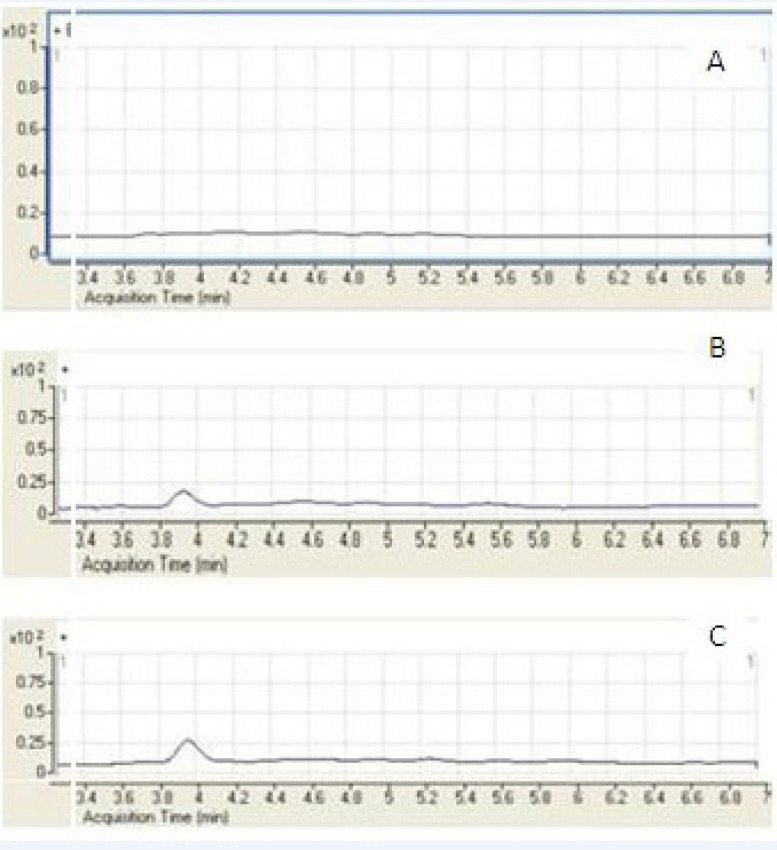
Representative SIM chromatograms of mebudioine. (A) Blank human plasma; (B) plasma spiked with mebudipine at the LLOQ of 5 ng/mL; (C) plasma sample from a volunteer 2.5 h after oral administration of 2.5 mg mebudipine.

A few representative chromatograms of mebudipine and carbamazepine are shown in Figure 3. The retention times of IS and mebudipine in total ion chromatogram were 2.86 and 3.82 min, respectively.

*Method validation*

*Selectivity*

The LC/ESI–MS method shows high selectivity because only selected ions from the analyte of interest are monitored. Chromatograms of the blank and the spiked human plasma samples (Figure 3) indicated no significant interferences at the retention time areas of the analyte. ESI positive MS spectra for mebudipine and IS were dominated by the [M+23]^+^ ions, *i.e. m*/*z *411 for mebudipine and 259 for carbamazepine. 

It was very important to investigate the matrix effects to develop a reliable and reproducible LC/ESI–MS method. Here, the matrix effect was evaluated by the following experiments: mebudipine and carbamazepine were spiked separately into human blank plasma as well as into the mobile phase as solvent. After being treated according to the procedure described in section of sample preparation, these samples were injected into LC/ESI-MS. No significant difference was observed between the peak area in chromatogram of spiked plasma samples and the peak area in the chromatogram obtained by injection of the solution of mebudipine and IS in mobile phase. It was also shown that no endogenous compounds significantly influenced the ionization of mebudipine and IS. 

*Linearity and LLOQ*

**Figure 4:**
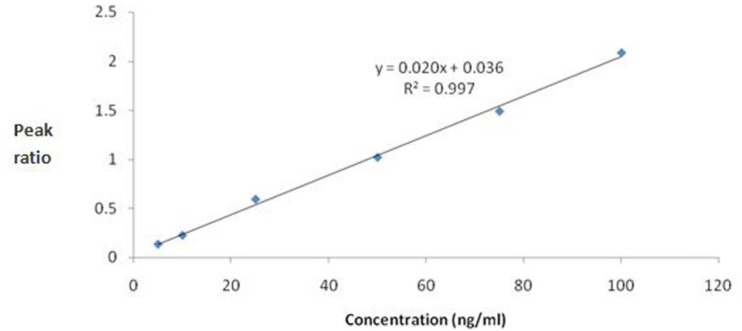
Calibration curve for mebudipine.

The standard calibration curves for mebudipine were linear over the concentration range of 5 –100 ng/mL (r^2^ ≥ 0.99). A typical regression equation for the calibration curves was Y=0.02X + 0.036, r^2^ = 0.997, where Y is the peak area ratio of mebudipine to IS, and X is the concentration of mebudipine in plasma (Figure 4).

The method reported here is very sensitive due to using optimum ESI-MS conditions and the advantages of LC/ESI-MS in the selected ion monitoring (SIM) mode. The lowest standard concentration in the calibration curve was considered as the LLOQ, which was 5 ng/mL. For LLOQ, the mean deviation percentage from the nominal concentration was 7.36% and precision was 1.3%. A good signal-to-noise ratio (10:1) was observed at the LLOQ indicating that the corresponding value could be reached. 

*Precision and accuracy*

The data of intra- and inter-day precision and accuracy for mebudipine from quality control samples are presented in Table 1. Coefficient of variation and error at all of the intra-day and inter-day assessment were less than 11%. The precision and accuracy of the present method conformed to the criteria for the analysis of biological samples according to the guidance of FDA where the CV determined at each concentration level is required to be not exceeding 15% (20% for LLOQ) and error within ±15% (±20% for LLOQ) of the actual value.

**Table 1:** Precision and accuracy for determination of mebudipine in human plasma (intra-day: n=5; inter-day: n=3 series per day, 3 days).

** Intra-day (** **n** **=5)**				**Inter-day (** **n** **=3** **)**		
Mebudipine (ng/mL)	mean ± S.D	CV%	error%	mean ± S.D	CV%	error%
5	5.12±0.54	10.54	2.4	5.01±0.21	4.19	0.2
50	49.53±4.64	9.37	0.93-	51.93±5.2	10.01	3.86
100	106.02±1.82	1.72	6.02	110.44**±**9.47	8.57	10.44

*Extraction efficacy*

Recovery of mebudipine was determined by comparing the peak area of the analyte extracted from the plasma with peak area obtained by the direct injection of pure standard analyte in mobile phase at three different concentrations containing low, middle and high concentration of mebudipin separately. The extraction recoveries of mebudipin from human plasma were 81.46%, 85.61% and 87.17% at concentrations of 10, 50 and 100 ng/mL, respectively (Table 2).

**Table 2:** Recoveries of mebudipine (n=3)

**Concentration (ng/mL) **	**Mebudipine (%)**
10	81.46**±**6.7
50	85.61±5.8
100	87.17±5.1

*Application to human study*

**Figure 5:**
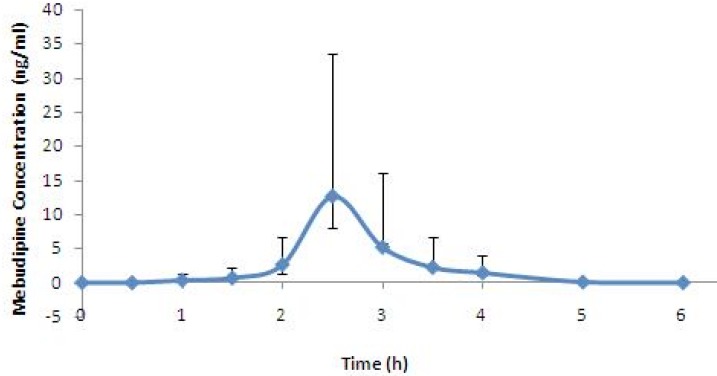
Mean plasma concentration vs time profile for mebudipine following oral dosing to human subjects

The developed method was successfully used for determination of plasma concentration of mebudipine after oral administration of single 2.5 mg tablet to healthy subjects. Mean serum concentration–time profiles mebdipine is shown in Figure 5. Peak plasma levels of mebudipine occurred after 2.5 h. After C_max_ was reached the plasma drug level declined rapidly with t_1/2_ of 0.6±0.07 h.

## Conclusions

In conclusion, we have developed and validated a rapid, sensitive and specific LC/ESI-MS method for determine of mebudipine in human plasma. According to our knowledge this is the first report on mebudipine detection in human plasma. Considering the fact that this validated method involves highly specific Mass detector it can be said that the current method is sensitive and reproducible enough to be used in pharmacokinetic studies.
